# Influence of surgical approach on complication risk in primary total hip arthroplasty: Systematic review and meta-analysis

**DOI:** 10.1080/17453674.2018.1438694

**Published:** 2018-02-16

**Authors:** Larry E Miller, Joseph S Gondusky, Atul F Kamath, Friedrich Boettner, John Wright, Samir Bhattacharyya

**Affiliations:** 1 Miller Scientific Consulting, Inc., Asheville; 2 Jordan-Young Institute, Virginia Beach; 3 Penn Medicine, Department of Orthopedic Surgery, Leonard Davis Institute of Health Economics, Philadelphia; 4 Hospital for Special Surgery, New York; 5 DePuy Synthes, Raynham, United States

## Abstract

**Background and purpose:**

Systematic comparisons of anterior approach (A) versus posterior approach (P) in primary total hip arthroplasty (THA) have largely focused on perioperative outcomes. In this systematic review with meta-analysis, we compared complication risk of A versus P in studies of primary THA with at least 1-year mean follow-up.

**Patients and methods:**

We performed a systematic review of prospective and retrospective studies with at least 1-year mean follow-up that reported complications of A and P primary THA. Complications included infection, dislocation, reoperation, thromboembolic event, heterotopic ossification, wound complication, fracture, and nerve injury. Random effects meta-analysis was used for all outcomes. Complication risk was reported as rate ratio (RR) to account for differential follow-up durations; values >1 indicated higher complication risk with A and values <1 indicated lower risk with A.

**Results:**

19 studies were included; 15 single-center comparative studies with 6,620 patients (2,278 A; 4,342 P) and 4 multicenter registries with 157,687 patients (18,735 A; 138,952 P). Median follow-up was 16 (12–64) months) with A and 18 (12–110) months with P. Anterior approach was associated with lower rate of infection (RR =0.55, p = 0.002), dislocation (RR =0.65, p = 0.03), and reoperation (RR =0.84, p < 0.001). No statistically significant differences were observed in rate of thromboembolic event (RR =0.59, p = 0.5), heterotopic ossification (RR =0.63, p = 0.1), wound complication (RR =0.93, p = 0.8), or fracture (RR =1.0, p = 0.9). There was a higher rate of patient-reported nerve injury with A (RR =2.3, p = 0.01).

**Interpretation:**

Comparing A with P in primary THA, A was associated with lower risk of reoperation, dislocation, and infection, but higher risk of patient-reported nerve injury.

The durability of total hip arthroplasty (THA) is excellent with 10-year survivorship exceeding 90% (Hailer et al. 2015, Makela et al. 2014). All standard approaches to the hip have been shown to be safe and effective, with certain advantages and disadvantages of each approach (Mjaaland et al. 2017). While the anterior approach (A) has been increasingly used in the United States, little is known about the safety of the A relative to other common surgical approaches. Several groups (Higgins et al. 2015, Meermans et al. 2017, Putananon et al. 2018) have performed systematic reviews comparing the A with the posterior approach (P) in primary THA. However, follow-up durations of the included studies varied widely, with most studies having less than 1-year follow-up. Comparative safety evaluation of these surgical techniques over a longer period is warranted. The purpose of this systematic review with meta-analysis was to compare the complication risk of A versus P in studies with at least 1-year mean follow-up.

## Methods

### Literature search and data extraction

In accordance with the PRISMA guidelines, we searched MEDLINE and EMBASE for comparative studies of primary THA performed using the A or P. Therapeutic search terms consisting of THA and total hip arthroplasty were combined with the following surgical approach-specific search terms: anterior, direct, posterior, posterolateral, and Smith-Petersen. We also manually searched the Directory of Open Access Journals (DOAJ), Google Scholar, and the reference lists of included papers and relevant systematic reviews. No language or date restrictions were applied to the searches. The final search was conducted on June 30, 2017.

Study eligibility was determined by 2 independent researchers (LM, DF). Disagreements were resolved by discussion. Main inclusion criteria included comparison of A versus P in primary THA, predominant diagnosis of osteoarthritis, mean follow-up duration at least 1 year, and extractable complication data. Titles and abstracts were initially screened to exclude review articles, commentaries, letters, case reports, and obviously irrelevant studies. Full-texts of the remaining articles were retrieved and reviewed. Studies were excluded if patients received revision or bilateral THA. When multiple studies included overlapping series of patients, only the study with the largest sample size was included. Data were independently extracted from eligible peer-reviewed articles by the same 2 researchers. Data discrepancies were resolved by discussion.

### Definitions and outcomes

When data were reported at multiple intervals during follow-up, the final value was extracted for analysis. Complications included infection, dislocation, reoperation (for any reason), thromboembolic event, heterotopic ossification, wound complication, fracture, and nerve injury. To account for differential follow-up durations, complication data were extracted by determining the number of events and then calculating the number of person-years in each group to determine incidence rates. Risk of bias in each study was assessed with the Cochrane Collaboration tool, which included evaluations of sequence generation, allocation concealment, blinding, incomplete outcome data, selective outcome reporting, and other sources of bias (Higgins et al. 2011).

### Data analysis

We assumed heterogeneous effects among studies a priori and conservatively applied a random effects model for all outcomes. Denominators were adjusted to include the number of patients or hips, as appropriate. The rate ratio (RR) was the effect size statistic of interest, which indicates the ratio of incidence rates (events per person-year) between A and P. A RR value >1 indicates higher complication incidence rate with A and a value <1 indicates lower complication incidence rate with A. For each complication, the RR and 95% confidence interval (CI) were calculated in each study and pooled among all studies. Inconsistency in complication risk among studies was quantified with the I^2^ statistic; values of ≤25%, 50%, and ≥75% represented low, moderate, and high inconsistency, respectively (Higgins et al. 2003). Publication bias was visually assessed with funnel plots (not shown) and quantitatively assessed using Egger’s regression test. Post hoc random effects meta-regression using the Knapp–Hartung method (Knapp and Hartung 2003) was performed to assess the possible influence of study design, median surgery year, inclusion of learning cases, and follow-up duration on complication risk. P-values were 2-sided with a significance level <0.05. Analyses were performed using Comprehensive Meta-analysis (version 3.3, Biostat, Englewood, NJ, USA).

### Funding and potential conflicts of interest

This work was supported by DePuy Synthes (Raynham, MA, USA). LM received a research grant from DePuy Synthes for data analysis. JW and SB are employees of DePuy Synthes. JG, AK, and FB declare no conflict of interest in this work.

## Results

### Study selection

After screening 340 records for eligibility, 19 studies were included in this review, including 15 single-center comparative studies with 6,620 patients (2,278 A; 4,342 P) and 4 multicenter registries with 157,687 patients (18,735 A; 138,952 P). Primary reasons for study exclusion included mean follow-up less than 1 year (27 studies), complications not reported (25 studies), and no comparison of A with P (20 studies) (Figure).

### Study and patient characteristics

This review included 4 randomized controlled trials, 1 prospective nonrandomized study, 10 retrospective studies, and 4 multicenter registries. Surgeries in each group occurred during the same period in 11 studies. In 7 studies, learning curve cases comprised some or all of the A group. Median follow-up duration was 16 months (range: 12–64 months) with A and 18 months (range: 12–110 months) with P. Comparing patients treated with A versus P, baseline patient characteristics were well matched for age (median 63 years per group), female sex (median 60% versus 58%), and BMI (median 28 per group) (Table 1). The primary risks of bias were attributable to inclusion of retrospective nonrandomized studies (Table 2).

**Table 1.** Study and patient characteristics

	Study	Treatment	Parallel treatment	Learning cases	Mean follow-up,months		Sample size^b^		Mean age,years		Female, %		Mean BMI	
Study	design^a^	period	period	included	A	P	A	P	A	P	A	P	A	P
Comparative studies:														
Balasubramaniam et al. 2016	RN	2006–2011	No	Yes	12	12	50	42	63	57	50	67	31	30
Barrett et al. 2013	RCT	2010–2011	Yes	No	12	12	43	44	61	63	33	57	31	29
Batailler et al. 2017	RN	2013–2015	Yes	Yes	14	14	201	101	72	74	65	65	26	28
Fransen et al. 2016	RN	2012	Yes	Yes	12	12	45	38	64	63	67	63	25	28
Luo et al. 2016	RCT	2014	Yes	No	14	14	52	52	62	64	67	58	23	24
Malek et al. 2016	RN	2010–2014	Yes	No	18	18	265	183	71	70	56	53	29	29
Newman et al. 2016	RN	–	NR	NR	24	24	235	120	63	59	54	57	29	34
Rathod et al. 2014	RN	2007–2011	No	No	16	30	286	293	62	61	55	57	26	26
Rodriguez et al. 2014	PN	2010	Yes	No	12	12	60	60	60	59	53	57	27	28
Sugano et al. 2009	RN	2005–2007	No	NR	24	24	33	39	56	57	88	92	23	23
Taunton et al. 2014	RCT	2012	Yes	No	12	12	27	27	62	66	56	52	28	29
Tripuraneni et al. 2016	RN	2012–2015	Yes	Yes	14	13	66	66	60	60	61	61	28	28
Tsukada and Wakui 2015	RN	2000–2009	No	NR	64	110	139	177	67	62	90	83	23	24
Watts et al. 2015	RN	2010–2014	NR	NR	12	12	716	3,040	64	62	51	51	29	30
Zhang et al. 2006	RCT	2002–2004	Yes	NR	20	20	60	60	61	63	58	53	–	–^c^
Registries														
Amlie et al. 2014	RN	2008–2010	Yes	No	24	30	421	421	67	66	69	64	–	–
Mjaaland et al. 2017	RN	2008–2013	Yes	Yes	52	52	2,017	5,961	67	65	67	65	–	–
Sheth et al. 2015	RN	2001–2011	No	Yes	36	36	1,851	31,747	65	66	60	58	28	29
Zijlstra et al. 2017	RN	2007–2015	No	Yes	40	40	14,446	100,823	–	–	68	68	–	–

A = anterior approach; P = posterior approach; NR = not reported

a Study design: PN = prospective nonrandomized; RCT = randomized controlled trial; RN = retrospective nonrandomized.

b Reported as number of patients or hips.

c All patients with BMI ≤27 kg/m^2^.

**Table 2.** Cochrane risk of bias assessment 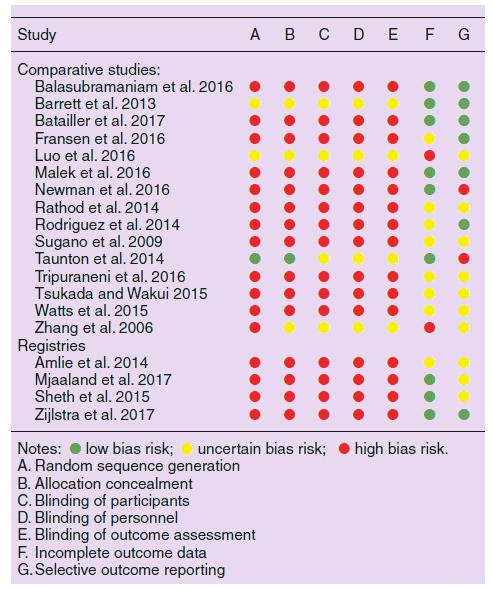

### Complications

The A was associated with lower rates of infection (RR =0.55, p = 0.002 from 7 studies), dislocation (RR =0.65, p = 0.03 from 11 studies), and reoperation (RR =0.84, p < 0.001 from 16 studies). In a subgroup analysis of infection, the rate of superficial (RR =0.47, p = 0.5) and deep infection (RR =0.23, p = 0.1) remained low with A, but neither was statistically significant. When explicitly reported, the most common reasons for reoperation were aseptic loosening, dislocation, fracture, and infection in the A group and dislocation, aseptic loosening, infection, and fracture in the P group. No statistically significant differences were observed in the rate of thromboembolic event (RR =0.59, p = 0.5 from 4 studies), heterotopic ossification (RR =0.63, p = 0.1 from 4 studies), wound complication (RR =0.93, p = 0.8 from 5 studies), or fracture (RR =1.0, p = 0.9 from 10 studies). Most fracture reports were of intraoperative periprosthetic fractures; however, type and time to fracture was not consistently reported. There was a higher rate of patient-reported nerve injury with A vs. P (RR =2.3, p = 0.01 from 2 studies). Nerve injuries were described as patient-reported sensory deficit (Luo et al. 2016) or patient-reported nerve injury with no distinction between sensory and motor involvement (Amlie et al. 2014). For each complication, heterogeneity among studies was low and publication bias was not evident (Table 3).

**Table 3.** Complication rates with anterior versus posterior approach in primary total hip arthroplasty

		Event rate per 100 person-years					
Outcome	Studies	A	P	Effect size Rate ratio (95% CI)^a^	p-value	Heterogeneity (I^2^), %	Publication bias (Egger’s p-value)
Infection	7	0.2	0.4	0.55 (0.38–0.80)	0.002	0	0.5
Thromboembolic event	4	0.5	1.1	0.59 (0.14–2.43)	0.5	0	0.2
Heterotopic ossification	4	1.5	2.3	0.63 (0.35–1.13)	0.1	0	0.3
Dislocation	11	0.2	0.2	0.65 (0.44–0.95)	0.03	17	0.5
Reoperation	16	0.6	0.7	0.84 (0.75–0.93)	< 0.001	0	1.0
Wound	5	1.7	1.9	0.93 (0.54–1.63)	0.8	0	0.4
Fracture	10	0.3	0.1	1.02 (0.75–1.38)	0.9	0	0.2
Patient-reported nerve injury	2	3.0	1.3	2.31 (1.22–4.39)	0.01	0	^b^

Notes: A = anterior approach; P = posterior approach.

a Rate ratio >1 indicates higher complication incidence rate with anterior approach; rate ratio <1 indicates lower complication incidence rate with anterior approach.

b Inadequate number of studies to calculate value.

### Post hoc meta-regression

Post hoc meta-regression was performed to assess the possible influence of study design, median surgery year, inclusion of learning cases, and follow-up duration on complication risk. No covariate was statistically significantly associated with the risk of any complication. In comparative studies, there was no statistically significant difference between A vs. P in the rate of any complication. In registries, the rate of patient-reported nerve injury was higher with A while the rates of infection and reoperation were lower with A (Table 4).

**Table 4.** Subgroup analysis of study design on complication rates with anterior versus posterior approach in primary total hip arthroplasty

	Comparative studies		Registries		
Outcome	Studies	Rate ratio (95% CI)^a^	Studies	Rate ratio (95% CI)^a^	p-value^b^
Infection	6	0.66 (0.16–2.7)	1	0.55 (0.37–0.80)	0.8
Thromboembolic event	4	0.59 (0.14–2.4)	0	–	–
Heterotopic ossification	3	0.58 (0.30–1.2)	1	0.81 (0.24–2.7)	0.6
Dislocation	8	0.55 (0.17–1.8)	3	0.74 (0.39–1.4)	0.7
Reoperation	12	1.03 (0.60–1.8)	4	0.83 (0.72–0.95)	0.5
Wound	5	0.93 (0.54–1.6)	0	–	–
Fracture	9	1.7 (0.79–3.7)	1	0.93 (0.66–1.3)	0.2
Patient-reported nerve injury	1	5.0 (0.24–104)	1	2.2 (1.2–4.3)	0.6

a Rate ratio >1 indicates higher complication incidence rate with anterior approach; RR <1 indicates lower complication incidence rate with anterior approach.

b Comparison of rate ratio in comparative studies versus registries, derived from Knapp– Hartung random effects meta-regression model.

## Discussion

We conducted a systematic review and meta-analysis of comparative studies of A versus P primary THA with at least 1-year mean follow-up. An anterior approach was associated with a lower risk of reoperation, dislocation, and infection, but higher risk of patient-reported nerve injury. No difference was seen in the rate of thromboembolic event, heterotopic ossification, wound complication, or fracture. While heterogeneity or publication bias was not evident for any outcome, the possibility of such influences cannot be ruled out given the small number of studies reporting each complication.

A criticism of the A in primary THA is the presence of a learning curve, during which complication rates may be elevated. In an analysis of over 5,000 THA procedures, 50 or more A procedures were required to overcome the learning curve (de Steiger et al. 2015). In a single-surgeon experience with the first 500 A cases, the most dramatic reduction in complication rates occurred after the first 100 cases (Hartford and Bellino 2017). We identified no substantial influence of learning case inclusion on complication rates in meta-regression although this analysis was limited since it was not possible to determine the percentage of the entire sample comprising learning cases.

We identified a higher rate of patient-reported nerve injury with A. In the study of Amlie et al. (2014), nerve injury was self-reported in 5.9% of A patients at 24 months follow-up and 3.3% of P patients at 30 months follow-up; however, there was no distinction between sensory or motor involvement. In another comparative study (Luo et al. 2016), sensory deficit was 3.8% with A and 0% with P at 14 months’ follow-up. While comparative nerve injury data were limited to these 2 studies, a high incidence of sensory deficit with A has been reported in other studies (Bhargava et al. 2010, Goulding et al. 2010). This is primarily attributable to likely iatrogenic injury of the lateral cutaneous femoral nerve. Despite the higher patient-reported nerve injury rate with A, long-term functional limitations or higher reoperation rates are unlikely with these events based on the findings from other studies (Bhargava et al. 2010, Goulding et al. 2010).

In a meta-analysis comparing A and P (Higgins et al. 2015), there were no group differences in risk of intraoperative fracture and lower risk of dislocation with A. More recently, a systematic review compared anterior, posterior, and lateral approaches in primary THA (Meermans et al. 2017). In that review, complications were not systematically evaluated although the authors concluded that there were similar rates of complications between surgical approaches. In a network meta-analysis of randomized controlled trials (Putananon et al. 2018), complication risk was reported to be lower with P vs. A (1.0% vs. 1.4%); however, specific complications were not described. Among these reviews, follow-up duration varied considerably and was generally less than 1 year. Key differences in our meta-analysis are inclusion of only those studies with mean follow-up of at least 1 year, reporting of multiple specific complications, and statistical adjustment to account for differential follow-up periods among studies.

Several aspects of our meta-analysis are novel including the longest duration follow-up of any A versus P review and a comprehensive assessment of complication rates. There are also several limitations. First, despite the longest mean follow-up of any review on this topic, it must be acknowledged that data derived from 16 (A) to 18 (P) months median follow-up must be considered preliminary. Further, while the RR statistic allows for group comparison of event rates on a common scale (per person-year), event rates that are non-constant with respect to time may complicate interpretation of these results. Second, while osteoarthritis was the predominant diagnosis in each study, reporting of THA indications was inconsistent and may have confounded outcomes. Third, due to the small number of studies reporting certain complications, some complication estimates reported in this review may change with the addition of data by future studies. Further, the influence of study design on complication rates should be interpreted cautiously given the small number of studies for subgroup comparisons. Fourth, complication reporting was generally inconsistent among studies. Adherence to standardized complication reporting guidelines would greatly improve data transparency and consistency in the THA literature. Fifth, no conclusions regarding complication risk with anterolateral or lateral approaches in THA may be derived from this review. Finally, 14 of 19 included studies were retrospective in nature, which are inherently prone to bias.

In summary, comparing A with P in primary THA, A was associated with a lower rate of reoperation, dislocation, and infection, but a higher rate of patient-reported nerve injury.  

Conception and design: LM, SB. Data collection: LM. Data analysis: LM. Writing the article: LM. Critical revision of the article: LM, JG, AK, FB, JW, SB

The authors would like to thank David Fay, PhD for assistance with literature review.

*Acta* thanks Johan Kärrholm and other anonymous reviewers for help with peer review of this study

**Figure:**
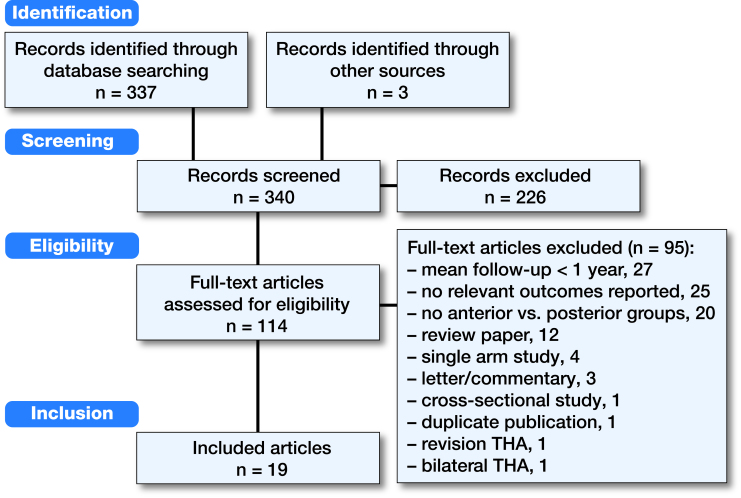
PRISMA study flow diagram.
